# Facial obstructions and baseline correction shape affective computing’s detection of emotion–behavior relationships

**DOI:** 10.3389/fpsyg.2026.1713462

**Published:** 2026-02-12

**Authors:** Anna Shepelenko, Vladimir Kosonogov, Anna N. Shestakova

**Affiliations:** 1 Institute for Cognitive Neuroscience, HSE University, Moscow, Russia; 2 Institute of Health Psychology, HSE University, Saint Petersburg, Russia

**Keywords:** affective computing, baseline correction, decision-making, donation behavior, electromyography, emotions, facial expression, methodological validity

## Abstract

**Introduction:**

Affective computing (AC) is increasingly used to study emotional processes underlying decision-making, yet its methodological validity in capturing spontaneous emotional responses and their behavioral relevance remains debated. In particular, it is unclear how facial obstructions and baseline correction affect the accuracy of AC-based facial emotion measures in capturing emotion–behavior relationships.

**Methods:**

The predictive validity of AC-based facial emotion measures was evaluated in charitable decision-making. Participants (*N* = 88) viewed dog images and made voluntary donations. Facial expressions were recorded using FaceReader (FR); in Group 1 (*n* = 43), facial electromyography (EMG) electrodes were also applied, while Group 2 (*n* = 45) had no electrodes. FR results were compared with EMG and self-report (SR) measures, and the effects of facial obstructions (presence of EMG electrodes) and baseline correction on AC accuracy were examined.

**Results:**

Donation behavior and SR emotion ratings were equivalent across both groups, whereas FR measurements differed. Corrugator EMG activity negatively correlated with SR and FR valence in both groups, but associations with FR were stronger when facial electrodes were absent, indicating that facial obstructions reduce AC accuracy. Across methods, stronger negative emotions were associated with higher donation amounts. FR valence and happiness correlated with donations across both samples, with stronger associations in the group without EMG electrodes; links with sadness and anger emerged only when facial expressions were unobstructed. Accounting for the prestimulus baseline improved convergence between FR and self-reported emotions and strengthened associations between FR-based valence and anger with donation behavior.

**Discussion:**

Results indicate that AC can capture prominent emotion–behavior relationships comparable to EMG and SR when recording conditions are optimal, but accuracy is reduced by facial obstructions and enhanced by baseline correction. These methodological factors are critical to consider in multimodal studies and research linking emotions to behavior.

## 1 Introduction

Emotional states permeate multiple levels of human cognition and behavior, influencing information processing (Lemerise and Arsenio, 2000), attitude formation and change (Petty and Briñol, 2015), decision-making (Lerner et al., 2015), and everyday social interaction (Van Kleef, 2009), among other psychological processes. Understanding emotional responses is therefore crucial across a wide range of domains, such as marketing (Pichierri et al., 2021), education (Zel et al., 2021), mental health (Drimalla et al., 2020), and psychology (Tyng et al., 2017). The influence of emotion on cognition and behavior is commonly understood as being mediated by appraisal processes, bodily responses, and expressive feedback mechanisms (Niedenthal et al., 2005; Scherer, 2009). Within appraisal theories, emotions arise from continuous evaluations of stimuli in relation to individual goals, while expressive components—such as facial muscle activity—reflect action tendencies that support behavioral regulation (Scherer, 2009). Complementarily, embodiment and affective neuroscience perspectives emphasize that facial and somatic feedback contributes to emotional experience and motivation rather than merely reflecting internal states (Niedenthal et al., 2005). Accordingly, even subtle, baseline-level variations in facial muscle activity have been shown to covary with affective processing and decision-related neural responses, including in the absence of overt emotional expressions (Adolphs, 2002; Davidson, 2004; Lench et al., 2011). From this perspective, facial expressions constitute an integral component of the mechanisms linking emotion to behavior. Consequently, factors that systematically distort facial signals—such as physical obstructions or inadequate baseline correction—may not only reduce measurement accuracy but also bias inferences about emotion–behavior relationships by altering the observable expressive input on which such inferences are based.

This theoretical emphasis on facial signals as meaningful components of emotional processing underscores the importance of how emotions are operationalized and measured in empirical research. Traditionally, emotion assessment has relied on self-report measures, manual coding of facial expressions, or EMG recordings, which can be time-consuming, intrusive, and susceptible to observer bias (Fridlund and Cacioppo, 1986; Mauss and Robinson, 2009). Building on the limitations of these traditional approaches, affective computing (AC) has been increasingly adopted as a standard method for assessing emotional states across various fields of scientific research (Fiala and Noussair, 2017; Magdin et al., 2021; Stillhart et al., 2025). Its growing popularity is largely justified because these methods do not require the installation of equipment on the human body or face, which significantly facilitates data collection and allows for ecologically valid research outside the laboratory, since any photograph or video recording can be analyzed using AC both during and after observations (Loijens and Krips, 2021).

Despite addressing some limitations of traditional emotion assessment methods, AC also introduces important ethical considerations and methodological challenges. Facial data constitute a sensitive category of personal information, and their collection and analysis require careful attention to informed consent, data protection, and responsible data handling (Barker et al., 2025; Mittelstadt et al., 2016). In addition, concerns have been raised regarding algorithmic bias in AC systems, as recognition accuracy may systematically vary across demographic groups due to imbalances in training data and model design (Barrett et al., 2019). Such biases are not only ethically relevant but also directly related to questions of validity, as systematic measurement error across individuals or groups can distort inferences about emotional processes and their behavioral correlates. These considerations underscore the importance of critically evaluating AC systems not only in terms of convenience and scalability but also with respect to methodological rigor and responsible use in research involving human participants.

At a methodological level, questions of validity in AC research are most commonly addressed through systematic validation studies focusing on recognition accuracy and convergence with established emotion measures (Beringer et al., 2019). Validation of AC systems typically relies on several methodological strategies, including comparison with manual coding by trained experts (Küntzler et al., 2021), performance on standardized image or video datasets (Lewinski et al., 2014), and convergence with physiological measures such as facial electromyography (EMG) (Westermann et al., 2024). These approaches primarily assess how well AC systems capture emotional information relative to established benchmarks.

Importantly, AC validity depends not only on the algorithms themselves but also on a range of additional factors. These include recording context characteristics—such as lighting, video quality, head position (Beringer et al., 2019), and the presence of facial obstructions (Kulke et al., 2020)—as well as methodological choices in data processing and analysis, including participant calibration and baseline procedures (Skiendziel et al., 2019). Validation studies also differ depending on whether emotions are elicited through voluntary, posed facial expressions or spontaneous, natural reactions (Höfling et al., 2021). These expression types vary in intensity and muscular activation and therefore pose distinct challenges for automated recognition (Cohn et al., 2007). Taken together, these considerations outline a multi-level framework for evaluating the validity of emotion assessment in AC, in which algorithmic performance, recording context, analytical procedures, and expression type jointly determine the quality of emotion estimates. Empirical findings relevant to this perspective are summarized below, highlighting validation approaches and influencing factors that have been systematically studied, those with mixed or inconsistent evidence, and those that remain underexplored.

The most widely used AC tools in research today, including FaceReader (FR; Loijens and Krips, 2021), Affectiva Affdex (McDuff et al., 2016), OpenFace (Baltrusaitis et al., 2016), recognize emotions from facial expressions by relying on the Facial Action Coding System (FACS; Ekman and Friesen, 1976). FACS is a descriptive framework that decomposes facial expressions into 46 action units (AUs), each corresponding to the activation of specific facial muscles (Ekman et al., 2002). Emotion recognition in FACS-based AC systems is based on identifying which AUs are active at a given moment and interpreting their combinations as discrete emotions. For example, sadness is typically associated with a pattern involving AU1 (inner brow raiser), AU4 (brow lowerer), and AU15 (lip corner depressor), with variation in activation strength reflecting emotion intensity (Ekman and Rosenberg, 1997). AC systems detect such AU patterns and map them onto emotion categories. Notably, most AC systems do not implement the full FACS repertoire. Instead, they rely on a reduced and system-specific subset of action units. For instance, FR version 8 detects 20 out of the 46 defined AUs and uses this information to classify six basic emotions (happiness, sadness, fear, anger, disgust, and surprise), and the neutral state, as well as valence and arousal (Loijens and Krips, 2021).

Other AC tools differ in the specific subset of AUs they detect and in the range of emotions or affective dimensions they infer based on this (Cohn and De La Torre, 2014; Namba et al., 2021). These implementation differences have tangible consequences for emotion recognition outcomes. Empirical comparisons demonstrate that, despite sharing a common FACS-based foundation, AC systems can yield substantially different results. A comparative analysis of nine widely used AC tools revealed marked variability in emotion recognition performance across platforms (Dupré et al., 2020), and systematic discrepancies have been observed even when identical stimuli are analyzed using different systems (Stöckli et al., 2018). Such differences have been attributed not only to variation in AU coverage but also to characteristics of the training data, including dataset size, representativeness, and annotation procedures (Namba et al., 2021). Consequently, AC systems are not directly interchangeable. Differences in AU coverage and model training result in system-specific performance, underscoring the need for cautious generalization and continued validation of individual AC tools.

A substantial body of empirical research has examined the validity of AC systems by comparing their outputs with manual facial coding performed by certified FACS experts, as well as by evaluating recognition accuracy on standardized facial expression image and video datasets (Küntzler et al., 2021; Lewinski et al., 2014; Namba et al., 2021; Skiendziel et al., 2019; Stöckli et al., 2018). Overall, the findings indicate that AC performs with high validity under controlled conditions—such as laboratory settings, studio lighting, and clearly expressed emotions—demonstrating accuracy comparable to manual FACS coding (Skiendziel et al., 2019). For instance, FR version 6.0 correctly identified 88% of target emotional labels in the Amsterdam Dynamic Facial Expression Set (ADFES; van der Schalk et al., 2011) and the Warsaw Set of Emotional Facial Expression Pictures (Lewinski et al., 2014). By contrast, in naturalistic conditions—such as film scenes or spontaneous emotional displays—AC systems tend to yield lower accuracy, particularly when AU activation is weak, emotions are subtle, or rare AUs are involved. In such cases, they often overestimate neutrality and fail to detect infrequent or low-intensity expressions (Küntzler et al., 2021; Namba et al., 2021; Skiendziel et al., 2019). In addition, recognition accuracy varies across specific emotions: while happiness is sometimes identified more accurately than by human subjects, fear tends to be recognized much less reliably, and confusions between fear, surprise, and anger can also occur (Küntzler et al., 2021; Namba et al., 2021; Skiendziel et al., 2019). In addition to these benchmark comparisons, a complementary line of validation research has examined the relationship between AC outputs and physiological measures of facial muscle activity, specifically EMG — widely regarded as a gold standard for objective assessment of facial expressions and associated emotional states (Fridlund and Cacioppo, 1986). During voluntary mimicry tasks, convergence has been observed between the electrical activity of the zygomaticus major (ZM) and corrugator supercilii (CS) muscles and automated assessments of joy and anger, respectively. This effect has been found using both Affdex (Kulke et al., 2020) and FACET (Beringer et al., 2019).

When emotions are elicited spontaneously or during passive viewing of affective stimuli, AC systems generally perform worse than EMG (Höfling et al., 2020, 2021). For example, FR 7.0 data collected during passive viewing of pleasant, neutral, and unpleasant images from the International Affective Picture System (IAPS; Lang et al., 2008) showed that FR valence consistently reflected EMG responses to positive stimuli, but correlations with negative stimuli were weak and latency was longer. Increased FR arousal scores corresponded to negative valence ratings, making this metric more sensitive to self-reported valence than to self-reported arousal (Höfling et al., 2020). Similarly, Affectiva Affdex captured joy-related expressions with delay and failed to register anger-related CS activity during passive viewing (Westermann et al., 2024). Direct comparisons of voluntary versus spontaneous facial mimicry confirmed that both FR 7.0 and EMG successfully differentiated expressions of joy, neutrality, and anger during active mimicry, whereas during passive viewing or emotion suppression, FR failed to detect changes while EMG remained highly sensitive (Höfling et al., 2021). Notably, more recent tools—including FR 9.0, Py-Feat 0.6.0, and OpenFace 2.2.0—showed a significant correlation between AU12 (lip corner puller), typically activated during smiling, and ZM EMG during passive viewing. However, all three AC systems were generally less sensitive and accurate than EMG, and, as observed in prior studies, showed a delayed response relative to EMG (Hsu and Sato, 2023).

As AC technologies continue to evolve, their ability to recognize emotions improves, and there is now substantial evidence supporting the convergence of AC outputs with manual FACS coding and EMG data when identifying emotions elicited by standardized stimuli and during voluntary mimicry (Höfling et al., 2020; Lewinski et al., 2014; Skiendziel et al., 2019). At the same time, under naturalistic conditions and for the detection of spontaneous emotional expressions, AC does not always reach the level of reliability sufficient to support robust conclusions regarding emotion-related hypotheses (Höfling et al., 2021; Hsu and Sato, 2023), highlighting the need for further refinement of AC algorithms.

Although studies have thoroughly compared the accuracy of various AC systems with EMG, manual coding, and self-reports, it remains unclear how consequential the identified limitations of AC are for research that is less concerned with precise emotion classification and more focused on linking emotional processes to behavior. This issue is particularly relevant for applied fields such as marketing (Caruelle et al., 2022) and education, where maintaining high ecological validity and natural participant behavior is essential. In such settings, the unobtrusiveness and ease of use offered by AC provide a clear advantage, yet a critical question remains: to what extent might the limitations of current technologies distort conclusions about causal links between emotion and behavior? Existing research has largely overlooked this issue, pointing to the need for applied comparative studies assessing the predictive power of different emotion-tracking methods in behavior-focused contexts.

In parallel to algorithm-focused validation efforts, individual empirical studies have examined how external recording conditions may affect the validity of AC data. For example, Beringer et al. (2019) analyzed how variables such as horizontal head angle, distance, brightness, and resolution impact the recognition of emotions depicted in standardized ADFES images (van der Schalk et al., 2011) and the detection of AUs in video stimuli from the Max Planck Institute (MPI; Troje and Bülthoff, 1996), using data from the FACET software. The results showed that manipulating all these variables within ranges typical of laboratory settings still allowed FACET to recognize emotions and AUs with high consistency with the corresponding reference emotion labels and AU annotations to the ADFES and MPI stimuli, with only resolution producing a significant effect on agreement indices (Beringer et al., 2019).

Beyond general recording parameters, the presence of objects on the face—such as beards, mustaches, glasses, EMG electrodes, or other equipment—can also impair the recognition performance of AC systems. This is particularly relevant for FACS-based software, as the presence of such objects may obscure certain AUs, limiting the system’s ability to accurately interpret emotional expressions, especially in passive viewing conditions where expressions are subtle. Illustratively, EMG electrodes placed over CS and ZM muscles may interfere with the detection of the corresponding AU4 and AU12, respectively, potentially distorting AC-based emotion classifications or leading to underestimation of emotion intensity. The presence of this effect may also help explain why simultaneous recordings using EMG and AC sometimes show insufficient convergence. From a methodological perspective, the importance of controlling facial obstructions is well established in facial EMG, where data quality critically depends on stable electrode–skin contact. Standard EMG protocols therefore emphasize careful preparation of recording sites, including cleaning and, when necessary, light abrasion, to ensure reliable signal acquisition (Fridlund and Cacioppo, 1986). In this context, facial objects that interfere with electrode placement or compromise electrode–skin contact are routinely considered during study design.

In contrast, in many studies validating AC by direct comparison with EMG, both methods have been recorded simultaneously without explicitly accounting for the potential impact of EMG electrodes on AC performance (Höfling et al., 2020, 2021; Westermann et al., 2024), despite evidence that their presence can reduce data validity (Kulke et al., 2020). Kulke et al. (2020), for instance, examined Affdex performance in recognizing posed happy, angry, and neutral expressions with and without EMG electrodes placed over the CS and ZM muscles. Compared to recordings without electrodes, recognition accuracy decreased, and neutral expressions were more frequently misclassified as negative (Kulke et al., 2020).

Crucially, this effect was demonstrated under conditions of voluntary facial mimicry, whereas the impact of EMG electrodes during spontaneous facial responses remains largely unexplored. Given that AC performance is generally lower for spontaneous than for posed expressions, unaccounted electrode interference may constitute a nontrivial source of bias in multimodal designs targeting spontaneous emotional processes. This effect was demonstrated under conditions of voluntary mimicry, whereas the impact of EMG electrodes during spontaneous facial responses remains largely unexplored. Given that AC performance is generally lower in spontaneous expression contexts, unaccounted electrode interference may constitute a nontrivial source of bias in multimodal designs targeting spontaneous emotional processes.

In addition to physical interference, data processing and recording protocols can introduce systematic variance into AC-based emotion estimates. In particular, objective measures of emotion require consideration of individual baseline states prior to stimulus presentation, as well as individual differences such as muscle tone or physiological reactivity. Facial EMG protocols address this through baseline correction, typically involving a short neutral phase recorded immediately before stimulus onset (Fridlund and Cacioppo, 1986), although in some protocols baseline is measured as a separate segment. During analysis, baseline activity is subtracted from stimulus-related muscle responses (Beringer et al., 2019; Höfling et al., 2020; Shepelenko et al., 2023), allowing clearer identification of stimulus-driven effects (Fridlund and Cacioppo, 1986) and improving consistency in interpreting findings.

In the case of emotion measurement using AC, no standardized approach has yet been established. To improve data accuracy, researchers currently employ either built-in or manual participant calibration, as well as prestimulus baseline procedures modeled after EMG protocols (Shepelenko et al., 2024a). Calibration typically involves capturing a neutral facial expression and adjusting subsequent data accordingly, which helps reduce the confounding effects of individual physiognomy (Loijens and Krips, 2021). In the context of voluntary mimicry, manual calibration of FR 7.0 data was found to slightly improve recognition accuracy, although the effect was small (Skiendziel et al., 2019). However, during passive viewing, calibration may be particularly relevant, because automatic facial expression analysis systems have been shown to exhibit reduced sensitivity to subtle or ambiguous expressions, a context in which individual facial morphology and features exert a relatively greater influence on outputs (e.g., Kim et al., 2023; Matsumoto and Hwang, 2014).

Prestimulus baselines, in turn, allow accounting for participants’ initial levels of facial muscle activity and ongoing affect-related variance prior to stimulus onset (Fridlund and Cacioppo, 1986; Rutkowska et al., 2024). Their use depends on the experimental design, but in studies where baseline affect may influence dependent variables—such as research on the relationship between emotions and decision-making—baseline recording is essential for accurate interpretation. Nonetheless, only a subset of studies using AC report implementing both baseline and calibration (Shepelenko et al., 2024a), while others mention only calibration (Kovalchuk et al., 2022; Skiendziel et al., 2019), only baseline (Fiala and Noussair, 2017), or neither (Maison and Pawłowska, 2017). This variation in data collection approaches complicates the generalization of findings and may account for observed discrepancies across studies. Moreover, published methodological discussions on AC rarely address the role of baseline correction in emotion assessment, making it difficult to determine the extent to which its absence may alter research outcomes.

Thus, prior research has demonstrated that affective computing systems can capture facial emotion signals with reasonable validity under controlled conditions, while also identifying multiple sources of distortion related to recording context, facial obstructions, and analytical procedures. At the same time, most validation efforts have concentrated on recognition accuracy or convergence with reference measures, leaving insufficiently explored whether these methodological limitations substantively affect conclusions in research that seeks to link emotional processes to behavior—particularly in contexts involving spontaneous emotional responding. This gap is especially consequential for behavior-focused and applied research, where AC is increasingly used as a primary, unobtrusive tool for emotion assessment under ecologically valid conditions. In such settings, methodological factors such as interference from facial EMG electrodes or the absence of baseline correction may not merely attenuate signal quality but may systematically bias inferences about how emotions guide behavior. Empirical evidence directly addressing the behavioral implications of these methodological choices, however, remains limited.

Accordingly, the present study examines the methodological conditions under which AC-based emotion measures derived from spontaneous facial expressions are used to predict behavior. The study focuses on charitable decision-making, a domain that is well established as being highly sensitive to emotional processes, including affective reactions to others’ need, empathic engagement, and affect-driven motivation to help (Sabato and Kogut, 2021; Shepelenko et al., 2024b; Small and Verrochi, 2009; van Doorn et al., 2017). This context therefore provides a theoretically meaningful test case for evaluating whether methodological distortions in emotion measurement translate into biased conclusions about emotion–behavior relationships.

Specifically, the study is guided by the following research questions:

1. To what extent do affective computing–based facial emotion measures capture spontaneous emotional responses, and how predictive are these responses of behavior compared to facial EMG and self-report measures in studies of emotion–behavior relationships?
2. To what extent does the presence of facial EMG electrodes alter affective computing–based emotion estimates during spontaneous facial responding, and how might such alterations bias inferences about emotion–behavior relationships in multimodal research designs?
3. To what extent does the implementation of baseline correction influence affective computing–based emotion estimates, and how does it affect the interpretation of emotion–decision relationships in research relying on spontaneous facial expressions?

By addressing these questions, the present study contributes to the affective computing and emotion–behavior literature in several ways. First, it extends existing validation research by shifting the focus from recognition accuracy to the behavioral consequences of methodological choices in AC-based emotion assessment. Second, by concentrating on spontaneous facial expressions rather than posed or voluntary mimicry, the study addresses an ecologically critical yet comparatively underexplored context in which AC is frequently applied. Third, the findings provide practical guidance for the design and interpretation of multimodal emotion research, particularly with respect to combining AC with EMG and implementing baseline correction procedures. More broadly, the results are relevant for applied domains such as psychology, education, healthcare, and other professional settings where emotion assessment must remain unobtrusive while supporting valid inferences about behavior, and they offer insights that may inform the further development of AC systems, including the integration of automated baseline handling to improve robustness in naturalistic research applications.

## 2 Methods

### 2.1 Participants

Eighty-eight healthy volunteers took part in the study, including 51 women, with the mean age of 23.6 years (SD = 5.1, range of 18–40). Participants were divided into two groups: Group 1 (FR + EMG) consisted of 43 individuals (25 women, mean age = 23.4 years, SD = 5.0, range 18–38), and Group 2 (FR) included 45 individuals (26 women, mean age = 23.9 years, SD = 5.3, range 19–40). The sample primarily comprised students and members of the general public recruited through online advertisements.

Due to technical issues during data acquisition, FR data were unavailable for three participants in Group 1, and EMG data were missing for an additional three participants. In Group 1, four participants wore thin-frame glasses, and two had short beards slightly covering the lower part of the face the lower part of the face. No participants in Group 2 wore glasses or facial hair. Across both groups, participants either wore no makeup or minimal everyday makeup, and no other facial piercings or objects were present beyond those already described.

All study procedures adhered to the Declaration of Helsinki and received approval from The HSE Committee on Interuniversity Surveys and Ethical Assessment of Empirical Research. Participants received 320 monetary units (MU) of the local currency at the beginning of the experiment as their only compensation for taking part in the study, equivalent to approximately 12 USD in 2023 after adjustment for purchasing power parity (World Bank, n.d.); no additional payments were provided. Participants were informed that they could donate all or part of the money during the experiment, retain the remainder, and that all donations were genuine and would be given to a charity disclosed after the experiment.

### 2.2 Material and design

The allocation of participants to two separate groups reflected methodological considerations of the study design. A between-subjects structure was selected to avoid the confounds inherent to repeated-measures approaches in this paradigm. Presenting the same images twice would have induced habituation and altered both emotional and donation responses, whereas using different image sets would have introduced systematic variability between conditions. The presence of EMG electrodes in only one condition would also have compromised comparability. Although individual differences in expressive behavior may contribute additional noise, this concern was partially mitigated by individual FR calibration. Given these factors, separating participants into two groups matched in age and gender distribution represented the most appropriate and methodologically robust solution.

Thirty-two images depicting dogs were selected from the Internet for the experiment. These images were chosen to elicit a range of emotional responses, including positive, negative, and neutral valence, and resembled those commonly used by pet charities to encourage donations for animals. This stimulus set has been previously employed in other studies (Shepelenko et al., 2023; Shepelenko et al., 2024a; Shepelenko et al., 2024b).

### 2.3 Procedure

**Figure 1:**
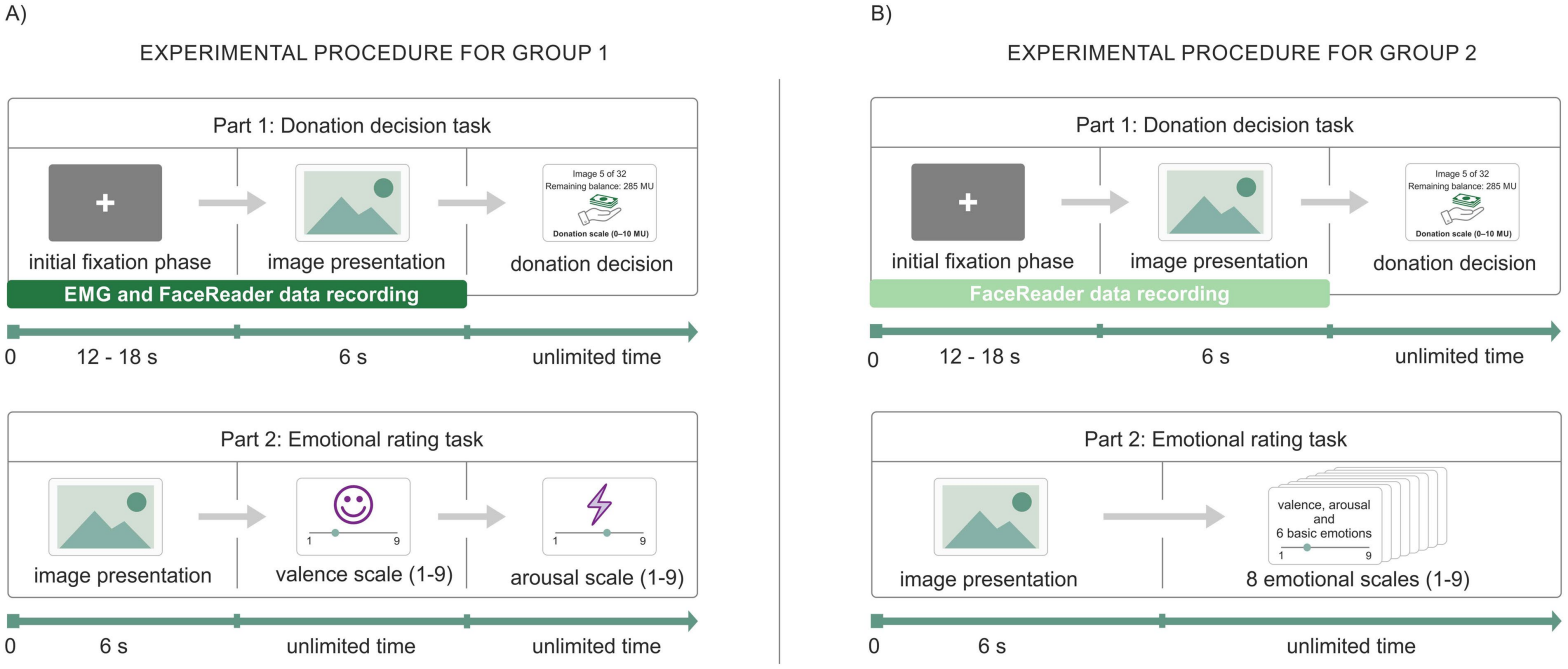
Experimental procedure for Group 1 and Group 2. **(A)** Experimental procedure for Group 1. **(B)** Experimental procedure for Group 2. Note: The experimental procedures for Groups 1 and 2 differed in the following ways: (1) EMG recording of ZM and CS muscles was conducted only for Group 1. (2) Participants in Group 1 rated only the valence (1 = very unpleasant, 5 = neutral, 9 = very pleasant) and arousal (1 = not at all aroused, 9 = very aroused) of each image, whereas participants in Group 2 rated valence, arousal, and six basic emotions (1 = not at all, 9 = extremely intense).

The experiment was conducted in a laboratory and consisted of two parts. In the first part, participants from both groups viewed each image for 6 s, then used a donation scale to allocate 0–10 monetary units (MU) from a total budget of 320 MU (Figure 1). The screen also displayed their current balance (e.g., 285 MU remaining) and the image number (e.g., 5 of 32). Thus, the maximum donation per animal was 10 MU, and participants knew there were 32 stimuli in total. The intertrial interval varied between 12 and 18 s to prevent emotional anticipation (Figure 1).

Stimuli were presented using a pseudo-randomized procedure. Five predefined presentation orders were constructed such that images depicting animals in similar conditions (e.g., visibly ill animals or puppies) did not appear consecutively. For each participant, different presentation orders were used in the first and second parts of the experiment, and the combinations of orders varied across participants. This procedure was implemented to reduce potential order effects and mitigate systematic influences of stimulus sequencing.

During the first part, facial expressions were recorded using FR for all participants. Additionally, in Group 1, EMG activity was recorded from ZM muscle, typically involved in raising the corners of the mouth during smiling (corresponding to AU12), and from CS muscle, typically involved in frowning by drawing the eyebrows together (corresponding to AU4) (Figure 1A). In the second part, participants again viewed images for 6 s and then rated their emotional state on a 9-point scale. Group 1 rated valence (negative to positive) and arousal (low to high) without time limits, while Group 2 rated valence, arousal, and six basic emotions (Figure 1). The use of multiple measurement modalities provided complementary assessments of emotional responses across participants.

### 2.4 Data collection and reduction

The experiment was conducted using PsychoPy software (Peirce et al., 2019). Physiological signals were recorded, amplified, and filtered via an ActiChamp data acquisition system (Brain Products, Germany) at a sampling rate of 500 Hz. EMG activity of CS and ZM muscles was measured using bipolar Ag/AgCl surface electrodes placed on the right side of the face. Before electrode placement, the skin was cleaned and degreased using alcohol wipes to reduce impedance. A small amount of conductive gel was applied to each electrode to ensure optimal signal quality. EMG data were processed using Chart5 software (ADInstruments). Stimulus onset was identified manually based on the signal from the photodiode channel, with an estimated temporal uncertainty of up to 100–200 ms. Raw EMG signals were subjected to digital band-pass filtering in the 10–350 Hz range, followed by full-wave rectification and integration with exponential decay (time constant 0.5 s). For further analysis, the mean values of the integrated signal were used.

For each stimulus presentation, EMG responses were calculated by subtracting the mean activity during the 1 s prestimulus interval from the average activity recorded during the 6-s display period. This baseline correction helped to control for the participant’s emotional state prior to stimulus exposure. A 1-s interval was selected because this duration is commonly used in psychophysiological research on affective EMG responses and provides a stable estimate of resting muscle activity while minimizing the influence of brief, spontaneous facial movements (e.g., Hazlett and Hoehn-Saric, 2000; Shepelenko et al., 2023). Using a longer baseline window might increase the chance of capturing transient, non-task-related facial activity, potentially reducing baseline stability.

Alongside EMG measurements, participants’ facial expressions were captured on video using a Logitech Webcam Brio (4,096 × 2,160 resolution) mounted above the computer monitor. Automatic analysis of these recordings was performed with FR software (version 8.0, Noldus Information Technology) at a sampling rate of 5 Hz for Group 1 and 10 Hz for Group 2, with 720p resolution (1,280 × 720). FR quantified two emotional dimensions—valence and arousal—as well as six basic emotions: happiness, sadness, anger, surprise, fear, and disgust, along with a neutral state. Valence scores ranged from −1 (highly negative) to 1 (highly positive), while arousal and the six basic emotions were scored from 0 (inactive) to 1 (fully active). Participant calibration involved selecting the frame with the lowest model error and non-neutral expressions for adjustment (Loijens and Krips, 2021). For each image presentation, emotional responses measured by FR were baseline-corrected using the same 1-s prestimulus interval to maintain methodological consistency. This interval has been previously applied in charitable-giving paradigms using FR (Shepelenko et al., 2024a). Emotional states were also calculated without baseline correction for Group 2 (FR) participants, allowing comparison of the two approaches to assess how baseline adjustment influences conclusions regarding emotion–donation relationships.

### 2.5 Data analysis

All analyses were conducted at the stimulus level (i.e., across individual images). Group comparisons were performed using either the Mann–Whitney U test or independent samples t-tests, depending on the type and distribution of the data. For normally distributed continuous variables with unequal variances, Welch’s t-test was applied. Statistical equivalence between groups was assessed with the Two One-Sided Tests (TOST) procedure using an 18% equivalence margin (Lakens, 2017). Relationships between variables were examined using Spearman or Pearson correlations computed on image-level means, with confidence intervals calculated via bootstrap resampling with 5,000 iterations for Spearman correlations and the Fisher r-to-z transformation for Pearson correlations. To test differences between correlation coefficients, bootstrap and paired bootstrap tests were applied with 5,000 iterations (DiCiccio and Efron, 1996). The Shapiro–Wilk test assessed normality for all variables. Unless otherwise specified, 95% confidence intervals (CI) were computed for all reported effects. Significance was set at *p* < 0.05, and multiple correlations were corrected using the false discovery rate method (Benjamini and Hochberg, 1995). All statistical analyses were performed in Python (v. 3.13.6) using Pandas, NumPy, SciPy, Statsmodels, Matplotlib, and Seaborn. Some Python scripts for data processing and analysis were generated or optimized with assistance from OpenAI’s ChatGPT (GPT-4.1) and Claude Opus 4.5. All scripts were manually reviewed and validated by the authors, with all utilized libraries explicitly documented, and are available at: https://osf.io/zn5eb.

## 3 Results

### 3.1 Comparison of behavioral, self-report, and FaceReader measures between groups

The results showed that participants’ donations, self-reported valence and arousal were almost entirely equivalent for both groups, as confirmed by the equivalence test (TOST, 18% margin): donations (*p*_low_ < 0.001, *p*_high_ = 0.034), SR valence (*p*_low_ = 0.028, *p*_high_ = 0.031), and SR arousal (*p*_low_ and *p*_high_ < 0.001), alongside difference tests: donations (*U* = 646.00, *p* = 0.170, *r* = −0.26), SR valence (*U* = 512.00, *p* = 1.000, *r* = 0.00), SR arousal (*U* = 647.00, *p* = 0.220, *r* = −0.26).

In contrast, the equivalence test for FR scores (TOST, 18% margin) across six basic emotions, valence, and arousal did not demonstrate equivalence between groups (all *p*s_low_ > 0.05). Additionally, difference tests conducted at the stimulus level (i.e., across images) revealed that certain emotions detected by FR were significantly weaker in Group 1 (FR + EMG) compared to Group 2 (FR): FR arousal *t*(46.25) = −8.21, *p* < 0.001, *d* = −2.05; FR anger *U* = 306.00, *p* = 0.041, *r* = 0.40; and FR sadness *t*(62) = −3.02, *p* = 0.007, *d* = −0.76.

Thus, self-reported measures were equivalent between groups, whereas FR scores were not equivalent and showed significant differences—Group 2 (FR) exhibited higher arousal, anger, and sadness than Group 1 (FR + EMG). Descriptive statistics, including means for all variables by group and overall averages for SR valence, arousal, and donations, are available in Supplementary Tables S1, S2.

### 3.2 Associations between EMG activity in Group 1 (FR + EMG) and emotional responses across groups

CS EMG in Group 1 (FR + EMG) was negatively correlated with SR valence in Group 1 (*r*_s_ = −0.87, *CI* [−0.94; −0.71], *p* < 0.001) and Group 2 (FR; *r*_s_ = −0.91, *CI* [−0.95; −0.80], *p* < 0.001). It also showed a negative correlation with FR valence in Group 1 (*r*_s_ = −0.48, *CI* [−0.72; −0.14], *p* = 0.021) and Group 2 (*r*_s_ = −0.83, *CI* [−0.93; −0.63], *p* < 0.001). This indicates that the stronger CS activation during image viewing, the more unpleasant emotions participants exhibited via self-report and FR scores. At the same time, the relationship between CS EMG and FR valence was significantly stronger in Group 2 (FR) compared to Group 1 (FR + EMG), *Δ*|*r*| = 0.35, bootstrap *CI* [−0.66; −0.07], *p* = 0.014 (here and throughout, Δ|r| denotes the difference between mean correlations across stimuli computed on the original data; confidence intervals and p-values were derived from bootstrap distributions of the corresponding differences). In contrast, correlations between CS EMG and SR valence did not differ significantly between groups, Δ|*r*| = 0.04, bootstrap *CI* [−0.08; 0.04], *p* = 0.557. For Group 1 (FR + EMG), EMG of CS was more strongly correlated with SR valence than with FR valence, *Δ*|*r*| = 0.39, bootstrap *CI* [0.19; 0.75], *p* < 0.001; however, for Group 2 (FR), these correlations did not differ significantly, Δ|*r*| = 0.08, bootstrap *CI* [−0.02; 0.27], *p* = 0.084.

CS EMG was also negatively correlated with FR happiness in Group 1 (FR + EMG; *r*_s_ = −0.55, −0.55, *CI* [−0.79; −0.16], *p* = 0.006) and Group 2 (FR; *r*_s_ = −0.80, −0.55, *CI* [−0.79; −0.16], *p* < 0.001), but this difference was not statistically significant, *Δ*|*r*| = 0.25, bootstrap *CI* [−0.40; 0.05], *p* = 0.126. EMG activity of the CS was positively associated with FR anger in Group 2 (*r*_s_ = 0.63, *CI* [0.34; 0.82], *p* < 0.001), but not in Group 1 (*p* > 0.05). CS activity in Group 1 also correlated with self-reported discrete emotions in Group 2, showing negative correlation with happiness (*r*_s_ = −0.82, *CI* [−0.91; −0.63], *p* < 0.001), and positive correlations with sadness (*r*_s_ = 0.90, *CI* [0.77; 0.95], *p* < 0.001), anger (*r*_s_ = 0.86, *CI* [0.73; 0.92], *p* < 0.001), disgust (*r*_s_ = 0.86, *CI* [0.73; 0.92], *p* < 0.001), fear (*r*_s_ = 0.89, *CI* [0.77; 0.93], *p* < 0.001), and surprise (*r*_s_ = 0.70, *CI* [0.52; 0.83], *p* < 0.001).

ZM EMG showed trend-level positive correlations with FR valence in Group 1 (*r*_s_ = 0.41, *CI* [0.09; 0.66], *p* = 0.069) and Group 2 (*r*_s_ = 0.39, *CI* [−0.01; 0.71], *p* = 0.083). However, after FDR correction, none of these associations remained significant. For all other self-reported or FR-registered emotions in both groups, correlations with ZM EMG were non-significant (all *p*s > 0.05).

### 3.3 Within-group associations between self-reported and FaceReader-derived emotions

Analysis of within-group correlations between self-reported and FR-registered emotions showed that SR valence correlated with FR valence in both Group 1 (*r*_s_ = 0.46, *CI* [0.14; 0.69], *p* = 0.030) and Group 2 (*r*_s_ = 0.87, *CI* [0.72; 0.94], *p* < 0.001), with the correlation being significantly stronger in Group 2 than in Group 1: *Δ*|*r*| = 0.41,bootstrap *CI* [−0.73; −0.19], *p* < 0.001. SR arousal and FR arousal did not show significant correlations in either Group 1 or Group 2.

Among basic emotions in Group 2 (FR), significant correlations were found between SR sadness and FR sadness (*r*_s_ = 0.55, *CI* [0.25; 0.75], *p* = 0.006), SR happiness and FR happiness (*r*_s_ = 0.87, *CI* [0.73; 0.93], *p* < 0.001), as well as SR anger and FR anger (*r*_s_ = 0.60, *CI* [0.29; 0.82], *p* = 0.001). All correlations and bootstrap-based comparisons associated with the analyses reported in Sections 3.2 and 3.3 are provided in the Supplementary materials (Supplementary Tables S3 and S4).

### 3.4 Relationships between emotional measures and donations

CS EMG in Group 1 correlated with donations from both Group 1 (*r*_s_ = 0.86, *CI* [0.68; 0.94], *p* < 0.001) and Group 2 (*r*_s_ = 0.90, *CI* [0.78; 0.95], *p* < 0.001), with no significant difference between correlations *Δ*|*r*| = 0.04, bootstrap *CI* [−0.15; 0.13], *p* = 0.855. ZM EMG in Group 1 did not correlate with donations in either Group 1 or Group 2 (all *p*s > 0.05). Thus, CS activity in Group 1 was similarly associated with donation amounts in both groups, with stronger CS activation corresponding to larger donations.

**Figure2:**
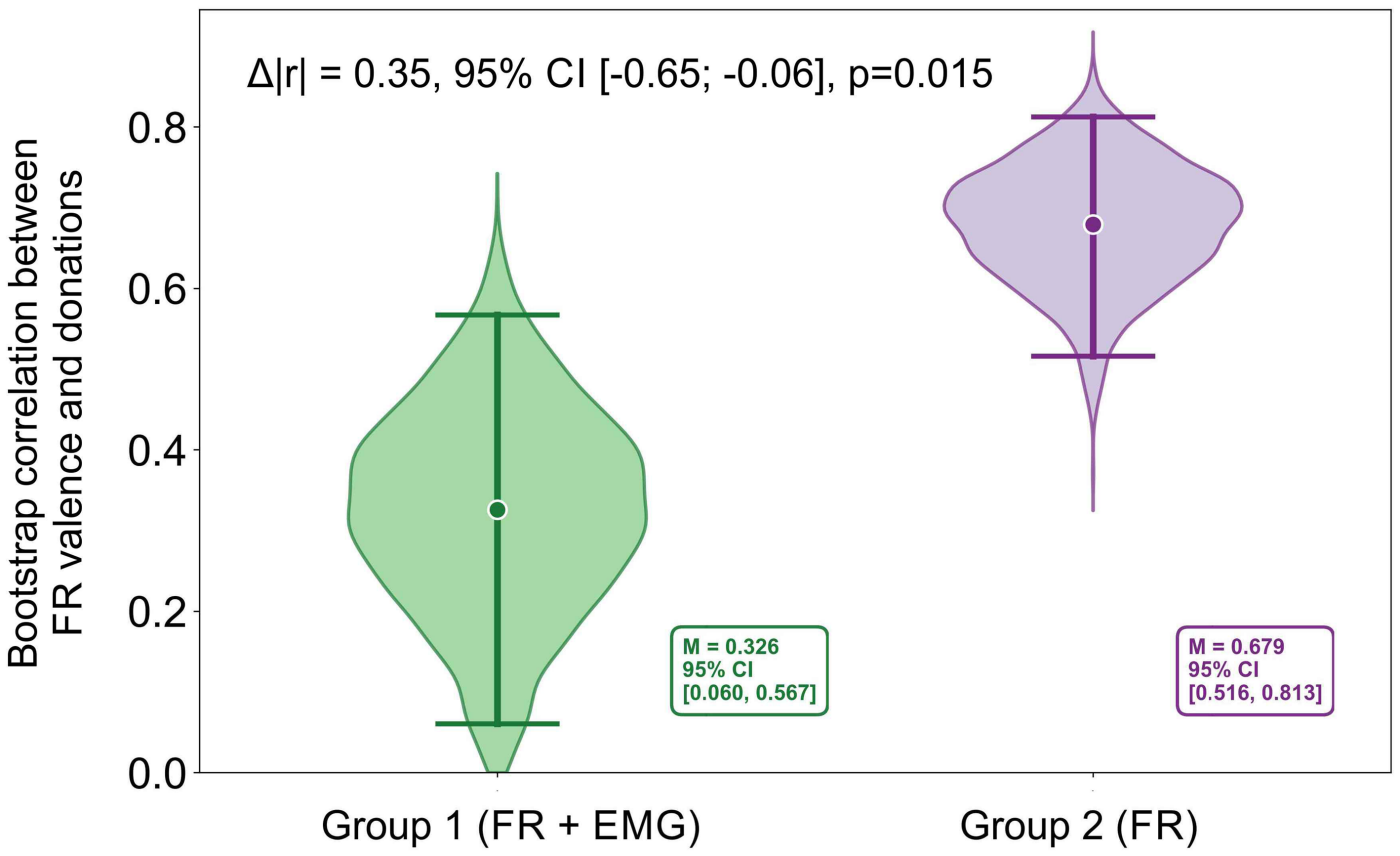
Bootstrap distributions of correlations between FR valence and donations in Group 1 (FR + EMG) and Group 2 (FR). In each iteration, subjects were resampled within each image, image-level means were computed, and correlations were calculated across images separately for each group. The annotated Δ|r| denotes the difference between bootstrap-based point estimates, whereas the Δ|r| reported in the text is computed from correlations averaged across stimuli in the original (non-bootstrap) data.

Donation amounts in Group 1 negatively correlated with SR valence in both Group 1 (*r*_s_ = −0.77, *CI* [−0.87; −0.57], *p* < 0.001) and Group 2 (*r*_s_ = −0.96, *CI* [−0.97; −0.89], *p* < 0.001), with a significant, albeit small, difference between correlations *Δ*|*r*| = 0.19, bootstrap *CI* [−0.28; −0.03], *p* = 0.017. Valence measured by FR correlated with donations in Group 1 (*r*_s_ = −0.51, *CI* [−0.76; −0.15], *p* = 0.012) and Group 2 (*r*_s_ = −0.83, *CI* [−0.90; −0.66], *p* < 0.001), with the association between donations and FR valence being stronger for data collected from Group 2 (without facial EMG sensors) Δ|*r*| = 0.32, bootstrap *CI* [−0.65; −0.06], *p* = 0.015 (Figure 2). SR arousal was positively correlated with donations in Group 1 (*r*_s_ = 0.48, *CI* [0.16; 0.69], *p* = 0.023), but not in Group 2 (*p* > 0.05). FR arousal did not show significant correlations with donations in either group (all *p*s > 0.05).

Among discrete emotions recorded by FR, FR happiness negatively correlated with donations in both Group 1 (*r*_s_ = −0.54, *CI* [−0.79; −0.16], *p* = 0.007) and Group 2 (*r*_s_ = −0.79, *CI* [−0.90; −0.57], *p* < 0.001), with a stronger effect observed in Group 2, although the difference between correlation coefficients did not reach significance *Δ*|*r*| = 0.25, bootstrap *CI* [−0.42; 0.04], *p* = 0.106. Additionally, donations correlated with FR sadness (*r*_s_ = 0.50, *CI* [0.19; 0.73], *p* = 0.014) and FR anger (*r*_s_ = 0.55, *CI* [0.24; 0.75], *p* = 0.006) in Group 2, but not in Group 1 (all *p*s > 0.05).

All discrete emotions collected as self-reports only for Group 2, showed that were associated with donations: SR sadness (*r*_s_ = 0.94, *CI* [0.85; 0.97], *p* < 0.001), SR happiness (*r*_s_ = −0.84, *CI* [−0.92; −0.66], *p* < 0.001), SR fear (*r*_s_ = 0.93, *CI* [0.85; 0.96], *p* < 0.001), SR disgust (*r*_s_ = 0.87, *CI* [0.72; 0.93], *p* < 0.001), SR surprise (*r*_s_ = 0.80, *CI* [0.60; 0.92], *p* < 0.001), and SR anger (*r*_s_ = 0.87, CI [0.72; 0.93], *p* < 0.001). According to self-reports, the more sadness, fear, anger, disgust, and surprise participants experienced, and the less happiness they reported, the more they were willing to donate to animal welfare. All correlations and bootstrap-based comparisons associated with the analyses reported in Sections 3.4 are provided in the Supplementary materials (Supplementary Tables S3 and S4).

Thus, for Group 1, donations correlated most strongly with CS EMG (*r*_s_ = 0.86, *CI* [0.68; 0.94], *p* < 0.001), with this correlation coefficient being not significantly higher than that of self-report *Δ*|*r*| = 0.09, bootstrap *CI* [−0.15; 0.13], *p* = 0.962, but significantly greater than correlations with FR valence *Δ*|*r*| = 0.35, bootstrap *CI* [0.12; 0.69], *p* = 0.0058, and FR happiness Δ|*r*| = 0.35, bootstrap *CI* [0.05; 0.47], *p* = 0.014. For Group 2, the highest correlations with donations were found for SR valence (*r*_s_ = −0.96, *CI* [−0.97; −0.89], *p* < 0.001), SR sadness (*r*_s_ = 0.94, *CI* [0.85; 0.97], *p* < 0.001), and SR fear (*r*_s_ = 0.93, *CI* [0.85; 0.96], *p* < 0.001). Differences between SR valence and SR sadness were not significant Δ|*r*| = 0.02, bootstrap *CI* [−0.04; 0.10], *p* = 0.462, nor were differences between SR valence and SR fear Δ|*r*| = 0.03, bootstrap *CI* [−0.02; 0.15], *p* = 0.161.

Among facial expression measures, the strongest correlation with donations in Group 2 was with CS activity from Group 1 (*r*_s_ = 0.90, *CI* [0.78; 0.95], *p* < 0.001); however, the association between donations and SR valence in Group 2 was significantly stronger Δ|*r*| = 0.06, bootstrap *CI* [−0.28; −0.02], *p* = 0.014, albeit with a small effect size. The correlation of SR valence with donations was also significantly stronger than that of donations with FR valence Δ|*r*| = 0.17, bootstrap *CI* [0.06; 0.35], *p* = 0.003.

### 3.5 Comparison of FaceReader data with and without baseline correction

The comparison of FR data processed with a 1-s baseline correction (BLC) and without baseline correction showed that data with the baseline correction correlated better with self-reported emotions. Specifically, SR valence correlated significantly strongly with FR valence with BLC (*r*_s_ = 0.87, [0.72; 0.93], *p* < 0.001) than with FR valence without BLC (*r*_s_ = 0.71, *CI* [0.43; 0.87], *p* < 0.001), albeit with a relatively small effect, Δ|*r*_s_| = 0.16, *CI* [0.04; 0.55], *p* = 0.020. Also SR happiness correlated more strongly with FR happiness with BLC (*r*_s_ = 0.87, *CI* [0.73; 0.93], *p* < 0.001) than with FR happiness without BLC (*r*_s_ = 0.79, *CI* [0.53; 0.92], *p* < 0.001); and SR sadness correlated similarly with FR sadness with BLC (*r*_s_ = 0.55, *CI* [0.25; 0.74], *p* = 0.001) and FR sadness without BLC (*r*_s_ = 0.56, *CI* [0.29; 0.74], *p* < 0.001). However, bootstrap analysis indicated that these differences did not reach significance (all *p*s > 0.05; confidence intervals overlapped). SR anger significantly correlated only with FR anger with BLC (*r*_s_ = 0.60, *CI* [0.29; 0.82], *p* < 0.001). Other self-reported emotions, including disgust, fear, surprise, and arousal, were not significantly related to FR scores regardless of baseline correction.

**Figure 3:**
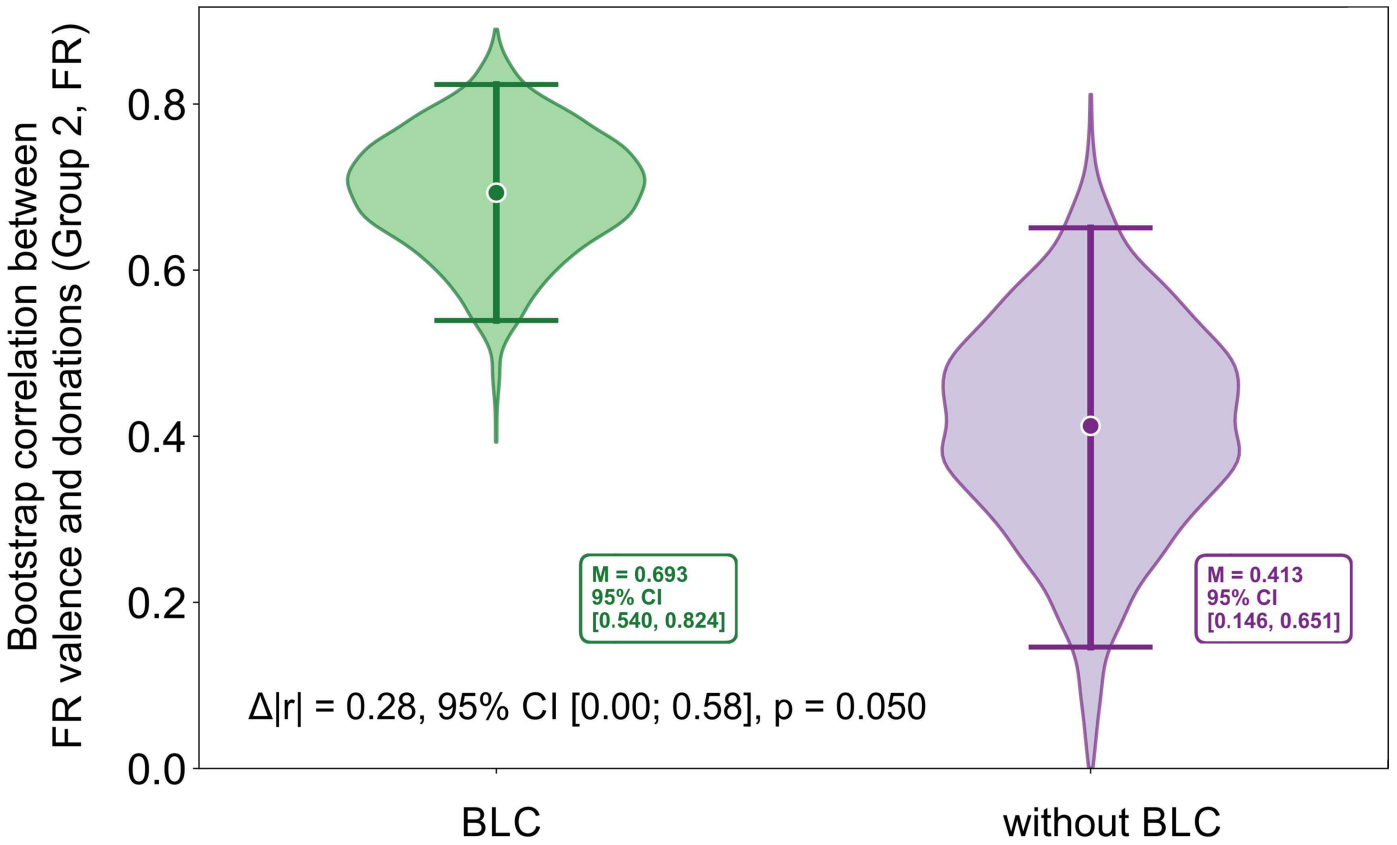
Bootstrap distributions of correlations between FR valence and donations in Group 2 (FR), comparing data processed with and without a 1-s baseline correction. In each iteration, bootstrap resampling was performed at the stimulus level, correlations were estimated separately for baseline-corrected and non-corrected data, and their difference was computed. The annotated Δ|r| denotes the magnitude of the difference between bootstrap-based correlation estimates, whereas the Δ|r| reported in the text is computed from correlations averaged across stimuli in the original (non-bootstrap) data.

Regarding associations with donations, FR data from Group 2 processed without baseline correction showed that FR valence without BLC, like FR valence with BLC, negatively correlated with donation amount (*r*_s_ = −0.65, *CI* [−0.82; −0.35], *p* < 0.001), though the correlation coefficient was significantly lower compared to FR valence with BLC (*r*_s_ = −0.83, *CI* [−0.90; −0.66], *p* < 0.001), Δ|*r*| = 0.18, bootstrap *CI* [0.00; 0.58], *p* = 0.050 (Figure 3).

As for discrete emotions, FR data with and without baseline correction show both similarities and differences. FR happiness without BLC also negatively correlated with donations (*r*_s_ = −0.66, *CI* [−0.83; −0.34], *p* < 0.001), only slightly weaker than FR happiness with BLC (*r*_s_ = −0.79, *CI* [−0.90; −0.57], *p* < 0.001); however, the difference between the correlation coefficients did not reach statistical significance: Δ|*r*| = 0.13, bootstrap *CI* [−0.07; 0.39], *p* = 0.171. FR sadness without BLC showed a similar correlation with donations (*r* = 0.49, *CI* [0.17; 0.72], *p* = 0.015) as FR sadness with BLC (*r* = 0.50, *CI* [0.19; 0.73], *p* = 0.014), as confirmed by the bootstrap test: Δ|*r*| = 0.01, bootstrap *CI* [−0.19; 0.45], *p* = 0.384. However, FR data without baseline correction did not show a significant association between donations and anger (*p* > 0.05), unlike the baseline-corrected scores (FR anger BLС *r* = 0.55, *CI* [0.24; 0.75], *p* = 0.006). All correlations and bootstrap-based comparisons associated with the analysis reported in Section 3.5 are provided in the Supplementary Materials (Supplementary Tables S5 and S6).

## 4 Discussion

The present study investigated how emotions elicited during passive viewing of images of homeless animals are captured by different assessment methods—self-report, facial EMG, and affective computing—and how these emotion measures relate to charitable donation behavior. Using a multimodal design, we examined both the convergence between emotion indicators derived from these methods and the extent to which methodological factors specific to affective computing, namely facial obstruction by EMG electrodes and the use of prestimulus baseline correction, influence emotion estimates and their behavioral correlates. The discussion therefore proceeds in three steps: first, by comparing how emotions measured with different methods relate to each other; second, by examining how these emotion measures are associated with donation behavior; and third, by addressing how baseline correction affects the interpretation of emotions and emotion–behavior links. Thus, these parts of the discussion provide answers to the study’s research questions.

### 4.1 Convergence between emotion measures across assessment methods

The initial analysis addresses the correspondence between emotional responses assessed with affective computing, facial EMG, and self-report, with particular attention to convergence across methods and potential differences between recording conditions, as such discrepancies may reflect methodological rather than substantive differences relevant for subsequent analyses linking emotions to donation behavior. Across methods, comparable results were obtained for valence: SR valence did not differ between groups, and CS EMG activity in Group 1 (FR + EMG) showed similar correlations with SR valence in both groups. While CS EMG and SR valence correlated with FR valence in both groups, these associations were significantly stronger in Group 2 (FR). Moreover, FR valence differed between groups, and in Group 1 (FR + EMG), CS EMG was more strongly associated with SR valence than with FR valence. No such difference was observed in Group 2 (FR). These findings suggest that although subjective valence ratings were similar across groups, FR valence data from Group 1 (FR + EMG) showed weaker convergence with SR and EMG data compared to Group 2. This may reflect reduced AC accuracy due to the partial obstruction of key action units by electrodes, consistent with previous research (Kulke et al., 2020).

Our results partly align with Höfling et al. (2020), who found good convergence between EMG and FR during passive viewing of positive IAPS images. However, unlike their study, we found no systematic bias in FR valence across stimulus valence categories, possibly due to differences in design (e.g., stimuli or FR version) or EMG-related artifacts. On the whole, FR valence measured without facial obstructions showed strong convergence with both CS EMG and SR valence. In contrast to Hsu and Sato (2023), we found no consistent link between ZM EMG and FR valence: ZM activity was only associated with FR valence in Group 1 (FR + EMG) and not with SR valence or FR valence in Group 2 (FR). This may reflect a lack of smile-eliciting stimuli (23 out of 32 images were rated below 5 on a 9-point scale; overall mean ratings ranged from 1.9 to 7.5).

SR arousal scores were equivalent across Groups 1 and 2, suggesting that participants assessed arousal similarly. However, FR arousal was significantly lower in Group 1 (FR + EMG) than in Group 2 (FR). No significant correlations between SR and FR arousal were found in either group. These findings suggest substantial limitations of FR in detecting arousal, consistent with Höfling et al. (2020), who also reported no significant association between SR and FR arousal. FR scores on discrete emotions were not equivalent across groups, with Group 2 (FR) showing higher mean levels of all basic emotions compared to Group 1 (FR + EMG). These differences were significant for sadness and anger. CS EMG correlated comparably with FR happiness in both groups, suggesting that the EMG electrodes did not substantially impair recognition of happiness. This aligns with previous findings that AC systems reliably detect happiness (Beringer et al., 2019; Kulke et al., 2020; Westermann et al., 2024), and in some cases even outperform FACS coders (Skiendziel et al., 2019).

EMG correlated significantly with FR happiness only in Group 2 (FR) and only before FDR correction. Overall, we did not find a consistent relationship between ZM activity and FR happiness, in contrast to prior studies (Hsu and Sato, 2023; Kulke et al., 2020). As with valence, this may be explained by a lack of clearly positive stimuli and a relatively small sample size. FR sadness and FR anger also correlated with CS EMG, but only in Group 2 (FR). This may indicate a masking effect of the EMG electrodes, which partially obscure AU4 in the vicinity of the glabellar region, a reliable marker of both anger and sadness. This obstruction likely reduces the accuracy of AC systems in detecting these emotions. A similar interference effect may account for earlier findings where simultaneous EMG and AC recordings failed to reveal a link between anger and CS activity (Westermann et al., 2024).

Finally, self-reported discrete emotions, collected only in Group 2 (FR), significantly correlated with FR happiness, sadness, and anger. This supports the notion that in the absence of facial obstructions, FR can effectively detect these emotions—partially consistent with previous research (Beringer et al., 2019; Kulke et al., 2020). More broadly, emotion assessment during passive viewing of emotive images of homeless animals showed that FR reliably detects valence, happiness, sadness, and anger. This is supported by strong convergence between FR data, CS EMG, and self-reports when FR was recorded without EMG electrodes on the face.

### 4.2 Method-dependent detection of emotion–donation relationships

Building on the observed convergence and discrepancies between emotion measures, the next step concerns the extent to which emotions assessed with different methods are linked to actual donation behavior. Examining these associations allows assessing whether methodological differences in emotion measurement translate into differences in inferred emotion–behavior relationships. Across the emotion assessment methods considered, a consistent pattern emerged: participants in both groups donated more when experiencing negative emotions, and donation amounts did not differ between groups. CS EMG correlated with donations similarly in both groups, while ZM activity was unrelated to donation amounts. Both SR and FR valence correlated with donation behavior, with stronger correlations observed in Group 2 (FR). These findings align with prior research (Haynes et al., 2004; Sabato and Kogut, 2021; Shepelenko et al., 2024b; Small and Verrochi, 2009). Differences in SR and FR valence across groups suggest that valence may have had a greater emotional impact on Group 2 (FR). At the same time, the pronounced group difference in the correlation between FR valence and donations indicates that EMG electrodes may impair AC’s ability to recognize emotional expressions, thereby affecting the interpretation of behavior-related motives. Donations were also associated with SR arousal in Group 1 (FR + EMG) but not in Group 2 (FR), which may be due to procedural differences between the groups.

Among FR-detected discrete emotions, donations correlated with FR happiness in both groups and with FR sadness and anger only in Group 2 (FR). These differences indicate that previously observed FR limitations in emotion recognition can affect the ability to detect emotion–behavior links: in Group 1 (FR + EMG), the presence of EMG electrodes likely reduced the intensity of detected emotions, preventing associations between sadness/anger and donations. However, happiness, which AC systems detect most reliably, was consistently associated with donation behavior.

Self-reported emotions showed that lower joy and higher anger, sadness, fear, surprise, and disgust were associated with greater willingness to donate. Given that the images depicted homeless dogs, some of which were injured, participants likely experienced mixed negative emotions simultaneously, which was reflected in their responses. In this context, we suggest the presence of the mere-measurement effect (Godin et al., 2010), which may have influenced emotion ratings. At the same time, correlations between donations and sadness, happiness, and anger were consistent across self-reports and FR, suggesting robust associations between these emotions and donation behavior, consistent with prior findings in charitable giving (Small and Verrochi, 2009; van Doorn et al., 2017). These results highlight the value of combining subjective and objective measures of emotion, as this approach helps reveal stable emotion–behavior links even when one method alone lacks precision. In Group 1 (FR + EMG), donations were best predicted by CS EMG and SR valence, while in Group 2 (FR), by SR valence, SR sadness, CS EMG, and FR valence.

Overall, the results indicate that negative emotional valence constitutes a robust predictor of charitable giving across measurement modalities, whereas the detection of discrete emotion–behavior links depends more strongly on the method used and the recording conditions. When facial expressions were unobstructed, affective computing yielded emotion–donation associations largely consistent with EMG and self-report, whereas methodological interference—such as the presence of EMG electrodes—attenuated these links, particularly for sadness and anger.

### 4.3 The role of prestimulus baseline correction in emotion–behavior interpretation

Beyond physical interference from EMG electrodes, the analyses further demonstrate that data processing choices—specifically baseline correction—constitute an additional methodological factor shaping the validity of AC-based emotion measures. When FR data were recorded without EMG-related occlusions, accounting for a prestimulus baseline systematically influenced both emotion recognition accuracy and the interpretation of emotion–behavior relationships. FR estimates without baseline captured valence reasonably well, but significantly less accurately than FR data corrected for baseline. Convergence between FR and self-reported happiness and sadness also improved after baseline correction, although these differences did not reach statistical significance. Notably, FR anger correlated with SR anger only when baseline was included. These findings complement earlier research showing that calibrating AC to participants’ neutral state can slightly improve emotion recognition accuracy (Skiendziel et al., 2019). However, unlike Skiendziel and colleagues, our results revealed significant differences in valence and anger when a baseline was included. This highlights that when recording AC, it is important not only to neutralize individual physiognomic effects through participant calibration but also to account for their baseline state using a prestimulus baseline, especially when it may affect the accuracy of result interpretation.

Baseline correction also affected the interpretation of links between emotions and donation behavior. Consistent with self-report data, FR valence with baseline showed significantly stronger correlations with donations than FR valence without baseline. FR happiness and sadness also correlated more strongly with donations after baseline correction, though the differences were not statistically significant. Importantly, FR anger only correlated with donations when baseline was accounted for. Together, these results indicate that the absence of baseline correction may not merely reduce measurement precision but can attenuate or obscure emotion–behavior relationships, thereby biasing substantive conclusions in behavior-focused research relying on AC.

## 5 Limitations

Several limitations of the present study should be acknowledged. The attachment method used for facial EMG electrodes required additional skin-colored adhesive tape to ensure signal stability throughout the experiment. While methodologically necessary, this procedure resulted in partial occlusion of facial regions relevant for affective computing, covering approximately 10–12 cm^2^ of the area above the eyebrow and on the cheek. Such occlusion likely introduced additional noise and reduced the visibility of key action units, particularly those associated with sadness and anger. Relatedly, although the two groups were matched on age and gender, they were not fully equivalent in terms of facial appearance: a small number of participants in Group 1 wore thin-frame glasses or had short beards, whereas no participants in Group 2 had facial hair or glasses. While these features were minimal and generally considered to have limited impact on AC performance, their unequal distribution nonetheless constitutes an additional source of measurement variability that may have contributed to group differences in AC-based emotion estimates. Future studies may benefit from more compact EMG equipment that can be securely attached without auxiliary supports, thereby reducing interference with automated facial analysis and improving comparability across recording conditions.

A further limitation concerns the study design. A between-subjects approach was adopted such that facial EMG and affective computing data were obtained from different participants when examining cross-method relationships with donation behavior. This design choice was methodologically motivated to avoid habituation effects, stimulus-related confounds, and incompatibilities between EMG and AC recordings within the same session. Nevertheless, comparing emotion–behavior associations across groups introduces additional variability attributable to individual differences in emotional expressivity, which—despite partial mitigation through individual calibration of affective computing outputs—remains a constraint of the present design. In addition, the absence of a consistent association between zygomaticus major activity and valence or happiness may reflect both the relatively modest EMG sample size and the emotional characteristics of the stimulus set, which predominantly elicited negative affect and provided limited opportunities for smile-related activation. Future research should therefore include larger samples and a broader range of positively valenced stimuli to further examine convergence between EMG and affective computing for positive emotions.

## 6 Contributions and implications

Beyond the specific empirical findings reported above, the present study makes several broader contributions to research on affective computing and multimodal emotion assessment. First, it demonstrates that the validity of AC-based emotion measures cannot be evaluated independently of the behavioral inferences drawn from them: methodological factors such as facial obstruction and baseline handling systematically shape not only emotion estimates but also conclusions about emotion–behavior relationships. Second, by focusing on spontaneous facial responding in a prosocial decision-making context, the study provides evidence that AC can support behaviorally meaningful inferences under ecologically relevant conditions, provided that key methodological constraints are addressed.

More generally, these results inform the design of multimodal emotion research by clarifying when AC can be meaningfully combined with other physiological measures and when such combinations may introduce bias rather than added value. The findings are therefore relevant for applied domains in which unobtrusive emotion sensing is required, including human–computer interaction, healthcare, and other field settings, where AC is often deployed without direct validation against behavioral outcomes. Finally, the observed sensitivity of AC-based measures to baseline correction highlights a concrete avenue for methodological standardization and future system development, suggesting that automated handling of baseline states may be critical for improving robustness in naturalistic and longitudinal applications.

## 7 Conclusion

The present findings allow drawing explicit conclusions with respect to the research questions formulated in the introduction. First, addressing the question of whether affective computing–based facial measures capture spontaneous emotional responses and predict behavior comparably to facial EMG and self-reports, the results show that—when facial expressions are unobstructed—AC reliably detects emotional valence and several discrete emotions and yields emotion–donation associations largely consistent with those obtained using EMG and self-report. Second, in line with the question concerning the impact of facial EMG electrodes on AC-based emotion estimates, the data demonstrate that facial obstruction selectively alters AC outputs during spontaneous facial responding, attenuating the detection of sadness and anger and weakening inferred emotion–behavior relationships. Finally, addressing the question of baseline correction, the findings indicate that accounting for a prestimulus baseline systematically improves AC-based emotion estimates and strengthens their associations with donation behavior, revealing behaviorally relevant effects that remain obscured without baseline adjustment. Together, these results provide converging evidence that methodological factors specific to affective computing—namely facial obstruction and baseline correction—play a critical role in shaping both emotion estimates and conclusions about emotion–behavior relationships.

## Data Availability

The datasets presented in this study can be found in online repositories. The names of the repository/repositories and accession number(s) can be found at: https://osf.io/zn5eb.

## References

[ref1] Adolphs R. (2002). Neural systems for recognizing emotion. Curr. Opin. Neurobiol. 12, 169–177. doi: 10.1016/S0959-4388(02)00301-X, 12015233

[ref2] Baltrusaitis T. Robinson P. Morency L. P. (2016). OpenFace: an open source facial behavior analysis toolkit. Proceedings of the 2016 IEEE Winter Conference on Applications of Computer Vision (WACV).

[ref3] Barker D. Tippireddy M. K. R. Farhan A. Ahmed B. (2025). Ethical considerations in emotion recognition research. Psychol. Int. 7:43. doi: 10.3390/psycholint7020043

[ref4] Barrett L. F. Adolphs R. Marsella S. Martinez A. M. Pollak S. D. (2019). Emotional expressions reconsidered: challenges to inferring emotion from human facial movements. Psychol. Sci. Public Interest 20, 1–68. doi: 10.1177/1529100619832930, 31313636 PMC6640856

[ref5] Benjamini Y. Hochberg Y. (1995). Controlling the false discovery rate: a practical and powerful approach to multiple testing. J. R. Stat. Soc. Ser. B Stat Methodol. 57, 289–300. doi: 10.1111/J.2517-6161.1995.TB02031.X

[ref6] Beringer M. Spohn F. Hildebrandt A. Wacker J. Recio G. (2019). Reliability and validity of machine vision for the assessment of facial expressions. Cogn. Syst. Res. 56, 119–132. doi: 10.1016/J.COGSYS.2019.03.009

[ref7] Caruelle D. Shams P. Gustafsson A. Lervik-Olsen L. (2022). Affective computing in marketing: practical implications and research opportunities afforded by emotionally intelligent machines. Mark. Lett. 33, 163–169. doi: 10.1007/S11002-021-09609-0

[ref8] Cohn J. F. Ambadar Z. Ekman P. (2007). “Observer-based measurement of facial expression with the facial action coding system” in Handbook of emotion elicitation and assessment. eds. Coan J. A. Allen J. J. B. (Oxford: Oxford University Press), 203–221.

[ref9] Cohn J. F. De La Torre F. (2014). “Automated face analysis for affective computing” in The Oxford handbook of affective computing. eds. Rafael C. Sidney D.’. M. Jonathan G. Arvid K. (Oxford: Oxford University Press), 131–150.

[ref10] Davidson R. J. (2004). What does the prefrontal cortex “do” in affect: perspectives on frontal EEG asymmetry research. Biol. Psychol. 67, 219–234. doi: 10.1016/J.BIOPSYCHO.2004.03.008, 15130532

[ref11] DiCiccio T. J. Efron B. (1996). Bootstrap confidence intervals. Stat. Sci. 11, 189–228. doi: 10.1214/ss/1032280214

[ref12] Drimalla H. Scheffer T. Landwehr N. Baskow I. Roepke S. Behnia B. . (2020). Towards the automatic detection of social biomarkers in autism spectrum disorder: introducing the simulated interaction task (SIT). NPJ Digit. Med. 3, 1–10. doi: 10.1038/S41746-020-0227-5, 32140568 PMC7048784

[ref13] Dupré D. Krumhuber E. G. Küster D. McKeown G. J. (2020). A performance comparison of eight commercially available automatic classifiers for facial affect recognition. PLoS One 15:e0231968. doi: 10.1371/JOURNAL.PONE.0231968, 32330178 PMC7182192

[ref14] Ekman P. Friesen W. V. (1976). Measuring facial movement. Environ. Psychol. Nonverbal Behav. 1, 56–75. doi: 10.1007/bf01115465

[ref15] Ekman P. Friesen W. V. Hager J. C. (2002). Facial action coding system: Manual. Salt Lake City, UT: Research Nexus.

[ref16] Ekman P. Rosenberg E. L. (1997). “What the face reveals: Basic and applied studies of spontaneous expression using the facial action coding system (FACS)” in What the face reveals: Basic and applied studies of spontaneous expression using the Facial Action Coding System (FACS). eds. Rosenberg E. L. Ekman P.. 3rd ed (Oxford: Oxford University Press).

[ref17] Fiala L. Noussair C. N. (2017). Charitable giving, emotions, and the default effect. Econ. Inq. 55, 1792–1812. doi: 10.1111/ecin.12459

[ref18] Fridlund A. J. Cacioppo J. T. (1986). Guidelines for human electromyographic research. Psychophysiology 23, 567–589. doi: 10.1111/J.1469-8986.1986.TB00676.X, 3809364

[ref19] Godin G. Sheeran P. Conner M. Delage G. Germain M. Bélanger-Gravel A. . (2010). Which survey questions change behavior? Randomized controlled trial of mere measurement interventions. Health Psychol. 29, 636–644. doi: 10.1037/A0021131, 20939639

[ref20] Haynes M. Thornton J. Jones S. C. (2004). An exploratory study on the effect of positive (warmth appeal) and negative (guilt appeal) print imagery on donation behaviour in animal welfare. Proceedings of the Marketing Accountabilities and Responsibilities: ANZMAC 2004 Conference, 29 November-01 December

[ref21] Hazlett R. L. Hoehn-Saric R. (2000). Effects of perceived physical attractiveness on females’ facial displays and affect. Evol. Hum. Behav. 21, 49–57. doi: 10.1016/S1090-5138(99)00036-7

[ref22] Höfling T. T. A. Alpers G. W. Gerdes A. B. M. Föhl U. (2021). Automatic facial coding versus electromyography of mimicked, passive, and inhibited facial response to emotional faces. Cognit. Emot. 35, 874–889. doi: 10.1080/02699931.2021.1902786, 33761825

[ref23] Höfling T. T. A. Gerdes A. B. M. Föhl U. Alpers G. W. (2020). Read my face: automatic facial coding versus psychophysiological indicators of emotional valence and arousal. Front. Psychol. 11:521353. doi: 10.3389/FPSYG.2020.01388 PMC7316962 32636788

[ref24] Hsu C. T. Sato W. (2023). Electromyographic validation of spontaneous facial mimicry detection using automated facial action coding. Sensors 23:9076. doi: 10.3390/S23229076, 38005462 PMC10675524

[ref25] Kim H. Küster D. Girard J. M. Krumhuber E. G. (2023). Human and machine recognition of dynamic and static facial expressions: prototypicality, ambiguity, and complexity. Front. Psychol. 14:1221081. doi: 10.3389/FPSYG.2023.1221081, 37794914 PMC10546417

[ref26] Kovalchuk Y. Budini E. Cook R. M. Walsh A. (2022). Investigating the relationship between facial mimicry and empathy. Behav. Sci. 12:250. doi: 10.3390/BS12080250, 35892350 PMC9330546

[ref27] Kulke L. Feyerabend D. Schacht A. (2020). A comparison of the Affectiva iMotions facial expression analysis software with EMG for identifying facial expressions of emotion. Front. Psychol. 11:492813. doi: 10.3389/FPSYG.2020.00329, 32184749 PMC7058682

[ref28] Küntzler T. Höfling T. T. A. Alpers G. W. (2021). Automatic facial expression recognition in standardized and non-standardized emotional expressions. Front. Psychol. 12:627561. doi: 10.3389/FPSYG.2021.627561, 34025503 PMC8131548

[ref29] Lakens D. (2017). Equivalence tests: a practical primer for t tests, correlations, and Meta-analyses. Soc. Psychol. Personal. Sci. 8, 355–362. doi: 10.1177/1948550617697177, 28736600 PMC5502906

[ref30] Lang P. J. Bradley M. M. Cuthbert B. N. (2008). International affective picture system (IAPS): Affective ratings of pictures and instruction manual. University of Florida, Gainesville, FL: Technical Report A-8.

[ref31] Lemerise E. A. Arsenio W. F. (2000). An integrated model of emotion processes and cognition in social information processing. Child Dev. 71, 107–118. doi: 10.1111/1467-8624.00124, 10836564

[ref32] Lench H. C. Flores S. A. Bench S. W. (2011). Discrete emotions predict changes in cognition, judgment, experience, behavior, and physiology: a meta-analysis of experimental emotion elicitations. Psychol. Bull. 137, 834–855. doi: 10.1037/A0024244, 21766999

[ref33] Lerner J. S. Li Y. Valdesolo P. Kassam K. S. (2015). Emotion and decision making. Annu. Rev. Psychol. 66, 799–823. doi: 10.1146/ANNUREV-PSYCH-010213-115043, 25251484

[ref34] Lewinski P. Den Uyl T. M. Butler C. (2014). Automated facial coding: validation of basic emotions and FACS AUs in facereader. J. Neurosci. Psychol. Econ. 7, 227–236. doi: 10.1037/npe0000028

[ref35] Loijens B. L. Krips O. (2021). FaceReader Methodology Note. Available online at: https://info.noldus.com/hubfs/resources/noldus-white-paper-facereader-methodology.pdf (Accessed January 21, 2026).

[ref36] Magdin M. Balogh Z. Reichel J. Francisti J. Koprda Š. György M. (2021). Automatic detection and classification of emotional states in virtual reality and standard environments (LCD): comparing valence and arousal of induced emotions. Virtual Reality 25, 1029–1041. doi: 10.1007/s10055-021-00506-5

[ref37] Maison D. Pawłowska B. (2017). Using the Facereader method to detect emotional reaction to controversial advertising referring to sexuality and homosexuality. Springer proceedings in business and economics, January 2017, 309–327.

[ref38] Matsumoto D. Hwang H. C. (2014). Judgments of subtle facial expressions of emotion. Emotion 14, 349–357. doi: 10.1037/A0035237, 24708508

[ref39] Mauss I. B. Robinson M. D. (2009). Measures of emotion: a review. Cognit. Emot. 23, 209–237. doi: 10.1080/02699930802204677, 19809584 PMC2756702

[ref40] McDuff D. Mahmoud A. Mavadati M. Amr M. Turcot J. El Kaliouby R. (2016). AFFDEX SDK: a cross-platform real-time multi-face expression recognition toolkit. Proceedings of the 2016 CHI Conference on Human Factors in Computing Systems, 07-12-May-2016. 3723–3726.

[ref41] Mittelstadt B. D. Allo P. Taddeo M. Wachter S. Floridi L. (2016). The ethics of algorithms: mapping the debate. Big Data Soc. 3, 1–21. doi: 10.1177/2053951716679679

[ref42] Namba S. Sato W. Osumi M. Shimokawa K. (2021). Assessing automated facial action unit detection systems for analyzing cross-domain facial expression databases. Sensors 21:4222. doi: 10.3390/S21124222, 34203007 PMC8235167

[ref43] Niedenthal P. M. Barsalou L. W. Winkielman P. Krauth-Gruber S. Ric F. (2005). Embodiment in attitudes, social perception, and emotion. Personal. Soc. Psychol. Rev. 9, 184–211. doi: 10.1207/S15327957PSPR0903_1, 16083360

[ref44] Petty R. E. Briñol P. (2015). Emotion and persuasion: cognitive and meta-cognitive processes impact attitudes. Cognit. Emot. 29, 1–26. doi: 10.1080/02699931.2014.967183, 25302943

[ref6001] Peirce J. Gray J. R. Simpson S. MacAskill M. Höchenberger R. Sogo H. . (2019). PsychoPy2: Experiments in behavior made easy. Behav. Res. Methods 51, 195–203. doi: 10.3758/s13428-018-01193-y 30734206 PMC6420413

[ref45] Pichierri M. Peluso A. M. Pino G. Guido G. (2021). Health claims’ text clarity, perceived healthiness of extra-virgin olive oil, and arousal: an experiment using FaceReader. Trends Food Sci. Technol. 116, 1186–1194. doi: 10.1016/j.tifs.2021.05.032

[ref46] Rutkowska J. M. Ghilardi T. Vacaru S. V. van Schaik J. E. Meyer M. Hunnius S. . (2024). Optimal processing of surface facial EMG to identify emotional expressions: a data-driven approach. Behav. Res. Methods 56, 7331–7344. doi: 10.3758/S13428-024-02421-4, 38773029 PMC11362446

[ref47] Sabato H. Kogut T. (2021). Happy to help—if it’s not too sad: the effect of mood on helping identifiable and unidentifiable victims. PLoS One 16:e0252278. doi: 10.1371/journal.pone.0252278, 34061880 PMC8168869

[ref48] Scherer K. R. (2009). The dynamic architecture of emotion: evidence for the component process model. Cogn. Emot. 23, 1307–1351. doi: 10.1080/02699930902928969

[ref49] Shepelenko A. Kosonogov V. Shestakova A. (2023). How emotions induce charitable giving. A psychophysiological study. Soc. Psychol. 54, 261–270. doi: 10.1027/1864-9335/A000513

[ref50] Shepelenko A. Shepelenko P. Obukhova A. Kosonogov V. Shestakova A. (2024a). The relationship between charitable giving and emotional facial expressions: results from affective computing. Heliyon 10:23728. doi: 10.1016/j.heliyon.2023.e23728, 38347906 PMC10859774

[ref51] Shepelenko A. Shepelenko P. Panidi K. Kosonogov V. Shestakova A. (2024b). How the emotions evoked by homeless pets induce online charitable giving. J. Philanthr. Mark. 29:e1842. doi: 10.1002/NVSM.1842

[ref52] Skiendziel T. Rösch A. G. Schultheiss O. C. (2019). Assessing the convergent validity between the automated emotion recognition software Noldus FaceReader 7 and facial action coding system scoring. PLoS One 14:e0223905. doi: 10.1371/JOURNAL.PONE.0223905, 31622426 PMC6797095

[ref53] Small D. A. Verrochi N. M. (2009). The face of need: facial emotion expression on charity advertisements. J. Mark. Res. 46, 777–787. doi: 10.1509/jmkr.46.6.777

[ref54] Stillhart A. Häfliger R. Takeshita L. Stadlinger B. Leles C. R. Srinivasan M. (2025). Screening for dental pain using an automated face coding (AFC) software. J. Dent. 155:105647. doi: 10.1016/J.JDENT.2025.105647, 39993552

[ref55] Stöckli S. Schulte-Mecklenbeck M. Borer S. Samson A. C. (2018). Facial expression analysis with AFFDEX and FACET: a validation study. Behav. Res. Methods 50, 1446–1460. doi: 10.3758/s13428-017-0996-1, 29218587

[ref56] Troje N. F. Bülthoff H. H. (1996). Face recognition under varying poses: the role of texture and shape. Vis. Res. 36, 1761–1771. doi: 10.1016/0042-6989(95)00230-8, 8759445

[ref57] Tyng C. M. Amin H. U. Saad M. N. M. Malik A. S. (2017). The influences of emotion on learning and memory. Front. Psychol. 8:1454. doi: 10.3389/FPSYG.2017.01454, 28883804 PMC5573739

[ref58] van der Schalk J. Hawk S. T. Fischer A. H. Doosje B. (2011). Moving faces, looking places: validation of the Amsterdam dynamic facial expression set (ADFES). Emotion 11, 907–920. doi: 10.1037/A0023853, 21859206

[ref59] van Doorn J. Zeelenberg M. Breugelmans S. M. (2017). The impact of anger on donations to victims. Int. Rev. Victimol. 23, 303–312. doi: 10.1177/0269758017710819

[ref60] Van Kleef G. A. (2009). How emotions regulate social life: the emotions as social information (EASI) model. Curr. Dir. Psychol. Sci. 18, 184–188. doi: 10.1111/J.1467-8721.2009.01633.X

[ref61] Westermann J. F. Schäfer R. Nordmann M. Richter P. Müller T. Franz M. (2024). Measuring facial mimicry: affdex vs. EMG. PLoS One 19:e0290569. doi: 10.1371/JOURNAL.PONE.0290569, 38165847 PMC10760767

[ref6002] World Bank. (n. d.). Development Indicators: Table 4.16. World Bank. Available online at: https://wdi.worldbank.org/table/4.16 (Accessed January 21, 2026).

[ref62] Zel S. Duman G. Kongar E. (2021). Improving online learning experience using facial expression analysis. IEEE Eng. Manag. Rev. 49, 71–81. doi: 10.1109/EMR.2021.3079840

