# Supplementary material for: Facial obstructions and baseline correction shape affective computing’s detection of emotion–behavior relationships
Source: Front Psychol. 2026 Feb 12;17:1713462. doi: 10.3389/fpsyg.2026.1713462 (PMC12935594; doi:10.3389/fpsyg.2026.1713462)
Supplement: Supplementary file 1 [file Data_Sheet_1.docx]

**Table S1. Descriptive statistics of Group 1 (FR + EMG) variables averaged across participants by stimuli**

|  | **Valid** | **Missing** | **Mean** | **Std_Deviation** | **Shapiro-Wilk** | ***p*-value of Shapiro-Wilk** | **Minimum** | **Maximum** |
| --- | --- | --- | --- | --- | --- | --- | --- | --- |
| Donations | 32 | 0 | 6.39 | 1.068 | 0.921 | 0.022 | 4.628 | 7.837 |
| Valence SR | 32 | 0 | 4.474 | 1.783 | 0.92 | 0.021 | 1.698 | 7.488 |
| Arousal SR | 32 | 0 | 5.285 | 0.602 | 0.965 | 0.381 | 3.977 | 6.419 |
| Valence FR | 32 | 0 | 0.004 | 0.012 | 0.976 | 0.669 | -0.025 | 0.026 |
| Arousal FR | 32 | 0 | 0.004 | 0.003 | 0.958 | 0.244 | -0.002 | 0.009 |
| Happiness FR | 32 | 0 | 0.001 | 0.005 | 0.919 | 0.019 | -0.01 | 0.015 |
| Sadness FR | 32 | 0 | -0.002 | 0.008 | 0.981 | 0.824 | -0.02 | 0.014 |
| Anger FR | 32 | 0 | -0.001 | 0.014 | 0.925 | 0.028 | -0.019 | 0.036 |
| Surprise FR | 32 | 0 | 0.0003 | 0.003 | 0.973 | 0.585 | -0.007 | 0.008 |
| Fear FR | 32 | 0 | -91.85 | 0.0004 | 0.976 | 0.679 | -93.05 | 0.0006 |
| Disgust FR | 32 | 0 | -26.13 | 0.001 | 0.974 | 0.63 | -0.002 | 0.532 |
| EMG Zg | 32 | 0 | 0.116 | 0.127 | 0.922 | 0.024 | -0.152 | 0.432 |
| EMG_Cor | 32 | 0 | 0.054 | 0.287 | 0.9 | 0.006 | -0.488 | 0.432 |

**Table S2. Descriptive statistics of Group 2 (FR) variables averaged across participants by stimuli**

|  | **Valid** | **Missing** | **Mean** | **Std. Deviation** | **Shapiro-Wilk** | ***p*-value of Shapiro-Wilk** | **Minimum** | **Maximum** |
| --- | --- | --- | --- | --- | --- | --- | --- | --- |
| Donations | 32 | 0 | 5.806 | 1.293 | 0.956 | .212 | 3.556 | 7.889 |
| Valence SR | 32 | 0 | 4.471 | 1.794 | 0.910 | .011 | 1.844 | 7.533 |
| Arousal SR | 32 | 0 | 5.005 | 0.698 | 0.965 | .371 | 3.711 | 6.267 |
| Happiness SR | 32 | 0 | 2.908 | 1.826 | 0.821 | < .001 | 1.067 | 6.578 |
| Sadness SR | 32 | 0 | 4.367 | 1.972 | 0.846 | < .001 | 1.133 | 6.622 |
| Anger SR | 32 | 0 | 1.840 | 0.723 | 0.902 | .007 | 1.022 | 3.222 |
| Surprise SR | 32 | 0 | 1.872 | 0.512 | 0.859 | < .001 | 1.311 | 3.178 |
| Fear SR | 32 | 0 | 1.751 | 0.668 | 0.870 | .001 | 1.022 | 3.578 |
| Disgust SR | 32 | 0 | 1.784 | 1.007 | 0.755 | < .001 | 1.022 | 4.844 |
| Valence FR | 32 | 0 | 0.005 | 0.037 | 0.890 | .004 | -0.053 | 0.109 |
| Arousal FR | 32 | 0 | 0.014 | 0.006 | 0.977 | .711 | 0.001 | 0.024 |
| Happiness FR | 32 | 0 | 0.015 | 0.026 | 0.848 | < .001 | -0.013 | 0.080 |
| Sadness FR | 32 | 0 | 0.005 | 0.009 | 0.950 | .146 | -0.010 | 0.026 |
| Anger FR | 32 | 0 | 0.007 | 0.012 | 0.955 | .205 | -0.022 | 0.026 |
| Surprise FR | 32 | 0 | 0.0003 | 0.001 | 0.966 | .404 | -0.002 | 0.003 |
| Fear FR | 32 | 0 | 0.0003 | 0.002 | 0.989 | .978 | -0.004 | 0.004 |
| Disgust FR | 32 | 0 | -0.0003 | 0.003 | 0.929 | .036 | -0.011 | 0.004 |
